# Inhibitory kappa B alpha expression in endometriosis

**DOI:** 10.4274/tjod.37801

**Published:** 2019-01-09

**Authors:** Fabio Barra, Lorenzo Ferro Desideri, Carolina Scala, Simone Ferrero

**Affiliations:** 1 Academic Unit of Obstetrics and Gynecology, IRCCS Ospedale Policlinico San Martino; Department of Neurosciences, Rehabilitation, Ophthalmology, Genetics, Maternal and Child Health (DiNOGMI), University of Genoa, Genoa, Italy

**Keywords:** Endometriosis, endometriotic implants, deep infiltrating endometriosis, inhibitory kappa B

## To the Editor,

We read with great interest the article by Arlier et al.^(1)^ entitled “Tumor necrosis factor alfa and interleukin 1 alfa-induced phosphorylation and degradation of inhibitory kappa B (IкB), alfa are regulated by estradiol (E_2_) in endometrial cells,” published in your journal. Interestingly, the authors demonstrated that ectopic endometrium was significantly characterized by less immunoreactivity for IкB, a cytoplasmatic inhibitor of the transcription factor nuclear factor (NF)-кB, and that E_2_ might modulate its expression. The rational of this study is based on evidence of the critical role of NF-кB, which mediates gene transcription of several protein involved in inflammation, angiogenesis, as well as in proliferation and reduced apoptosis of endometriotic cells. A previous study reported that an excessive activation of NF-кB might be present in endometriotic implants of women affected by endometriosis^(2)^. For this reason, it might represent an interesting target for treating this benign chronic hormonal-dependent disease^(3)^. Although the authors should be congratulated for their laboratory findings, we would like to discuss some methodologic concerns of their study. In the material and methods, the authors described that the ectopic endometrial samples for western blot and immunocytochemical analysis were obtained from 6 women with endometriosis. First, the authors should add information on the severity of endometriosis for each patient in accordance with American Society of Reproductive Medicine classification. More importantly, the authors did not report from which sites the endometriotic implants were obtained, and in particular, if they originated from peritoneal nodules, ovarian endometriomas or deep infiltrating endometriosis (DIE) nodules. In general, it would be of particular interest to know if the activity of IкB and the effect of E_2_ on its expression would be different in implants originating from these three distinguished phenotypes of endometriosis, which probably have different pathogeneses^(4)^. Regarding this aspect, it has been previously described that nodules of DIE may have higher proinflammatory reaction, higher vascularization, as well a higher density of nerve fibers, which may be responsible for a more aggressive clinical behavior^(5)^. Nevertheless, the results of the study by Arlier et al.^(1)^ are innovative and promising. Thus, in the near future, new studies are needed to better clarify the role IкB in inflammatory pathway of endometriotic cells. More importantly, it could be advisable to better understand if this protein may represent a suitable molecular target for a chronic medical therapy, which needs to balance clinical efficacy with cost and tolerability.
